# Digital tissue and what it may reveal about the brain

**DOI:** 10.1186/s12915-017-0436-9

**Published:** 2017-10-30

**Authors:** Josh L. Morgan, Jeff W. Lichtman

**Affiliations:** 1 Department of Ophthalmology and Visual Sciences, Neuroscience, Washington University School of Medicine, St. Louis, MO 63108 USA; 2 Department of Molecular and Cellular Biology, Center for Brain Science, Harvard University, Cambridge, MA 02138 USA

## Abstract

Imaging as a means of scientific data storage has evolved rapidly over the past century from hand drawings, to photography, to digital images. Only recently can sufficiently large datasets be acquired, stored, and processed such that tissue digitization can actually reveal more than direct observation of tissue. One field where this transformation is occurring is connectomics: the mapping of neural connections in large volumes of digitized brain tissue.

## From drawings to digitization: the emergence of images as data

**Fig. 1.**
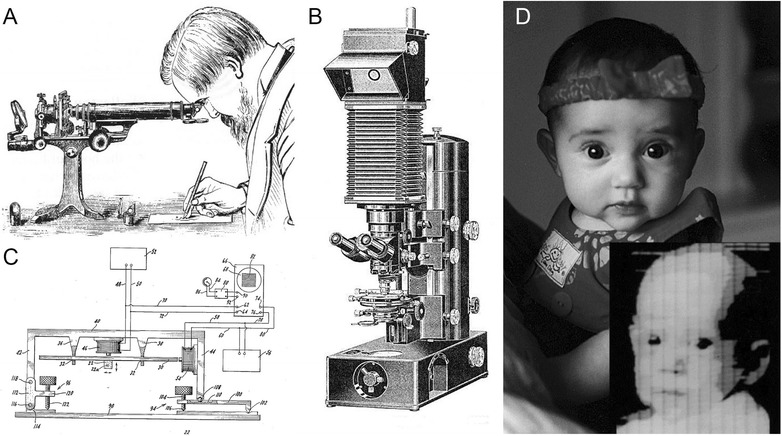
Changes in the way researchers render biological images. **a** The camera lucida is an optical device that projects a semitransparent image of a microscopic field of view onto the same plane as a sheet of drawing paper. Using this device, a scientist can trace exactly what they see in the microscope onto paper (illustration from 1857 catalogue of Messrs Ross). **b** Once photography was invented, it was obvious that microscopes should be equipped with a more automated way of generating a lasting rendering of what is in the field of view of a microscope objective. Shown here is the Zeiss Ultraphot I, the company’s first commercial photomicroscope (courtesy of Carl Zeiss Microscopy GmbH). **c** The first scanning confocal microscope as described by Marvin Minsky in his patent of 1957 [28]. This device used electromagnets (see labels 46 and 54) to move a sample (11a) in a raster pattern. The sample was illuminated with a focused spot of light and the return reflected light passed through a pinhole and was detected by a photomultiplier tube (PMT). The analog output of the PMT was displayed on an oscilloscope. **d** The first digital image shows Walden Kirsch in 1957 (*lower right*), son of the team leader that built the first image scanner [29]. The image is the sum of two binary scans set at different thresholds to produce approximate gray levels. This panel also shows a digital camera image that Walden himself took 40 years later of his own daughter at much higher resolution than the 176 × 176 pixels of the image of him and 24 bit depth rather than the binary (1 bit) depth. © 1998 IEEE. Reprinted, with permission, from Kirsch RA. SEAC and the start of image processing at the National Bureau of Standards. IEEE Ann History Comput. 1998;20(2);7–13; all rights reserved

In terms of imaging the world, the transition from painting to photography was a profound step as the image became, for the first time, an objective rendering of the world. Nowhere was this change more evident than in microscopy. In the first centuries after microscopes were built, scientists drew the new and previously invisible world based on their impressions. As a result, scientists as formidable as Golgi and Cajal could look at the same kind of material and report different things. This interpretive problem was partly overcome by the invention of the camera obscura and later the camera lucida, which allowed microscopists to trace exactly what was being viewed (Fig. 1a). But the skill of the tracer was still an obstacle to getting reliable information.

The development of photosensitive silver halide crystals as a medium for the capture and storage of visual information (Fig. 1b) marked a turning point in biological science. It allowed many people, who might disagree, to at least be interpreting the exact same data. Photography flourished in the last century and photo-microscopy became a central feature of nearly all publications related to the biology of cells—which is a vast literature.

The advent of television marked the birth of the ‘electronic’ image. In microscopy, light amplifying intensifiers that had been used in the military for low light imaging were combined with TV style cameras and high signal to noise ratio methods of fluorescence to render cellular events over time where the absolute intensity of the specimen was, until then, limiting. The analogue electronic image was a profound advance as it could be adjusted, for example, to change the image’s contrast or gamma in reproducible ways [1]. It also marked the birth of synthetic images where an optical path that produced a ‘real’ image was no longer a requirement. Thus, the scanning electron microscope, Minsky’s initial invention of the confocal, or Roberts and Young’s invention of the flying spot microscope all produced microscopy images by rendering serially obtained image data from a single point detector on a spatially distributed device like a TV monitor (Fig. 1c) [2, 3]. This scanning was an important advance because the image was now a pointillistic series of data values, each independent and measurable separately, the harbinger of true digital microscopy. This approach allowed the first digital images to be acquired (Fig. 1d)

Beginning in the 1990s, commercialization of computers and the exponential increase in their power brought about another revolution in biological imaging, one that is still going on and one that we think is as important as the transition from drawing to photography. Electronic imaging devices can now stream large amounts of image data directly to hard drive storage. Image data *acquisition* has thus become separated from the *rendering* of images with profound consequences.

One of these consequences is that much larger amounts of image data can be acquired than was possible with film. This particular kind of ‘big data’ is already having a big effect on biological microscopy. Indeed we are reaching the point where imaging, and storage and retrieval of digital image data, is so easy that it justifies a *shoot first ask questions later* approach to microscopy. As a consequence the work style of microscopists will change. Rather than searching tissue samples to find example images to make a particular point, the vast data of digitized biological specimens will be mined after, sometimes long after, image acquisition. These large data sets are also shareable, giving potentially any interested party access to entire tissues rather than just type-example images. Mass collection and mass distribution of biological data offer unprecedented opportunities for both reaching consensus and collaborative examination of data to detect complex or rare patterns that would otherwise be impossible to find.

We believe large volumes of three-dimensional digital image data will be especially useful for study of the cellular organization of the brain, the most complex tissue known. Because spatially extended neuronal networks are the basis of the brain’s functions, describing such networks requires access to digital versions of large tissue volumes but at resolutions sufficient to resolve subcellular synaptic details, in other words, big data.

## A big data view of the brain: connectomes

**Fig. 2.**
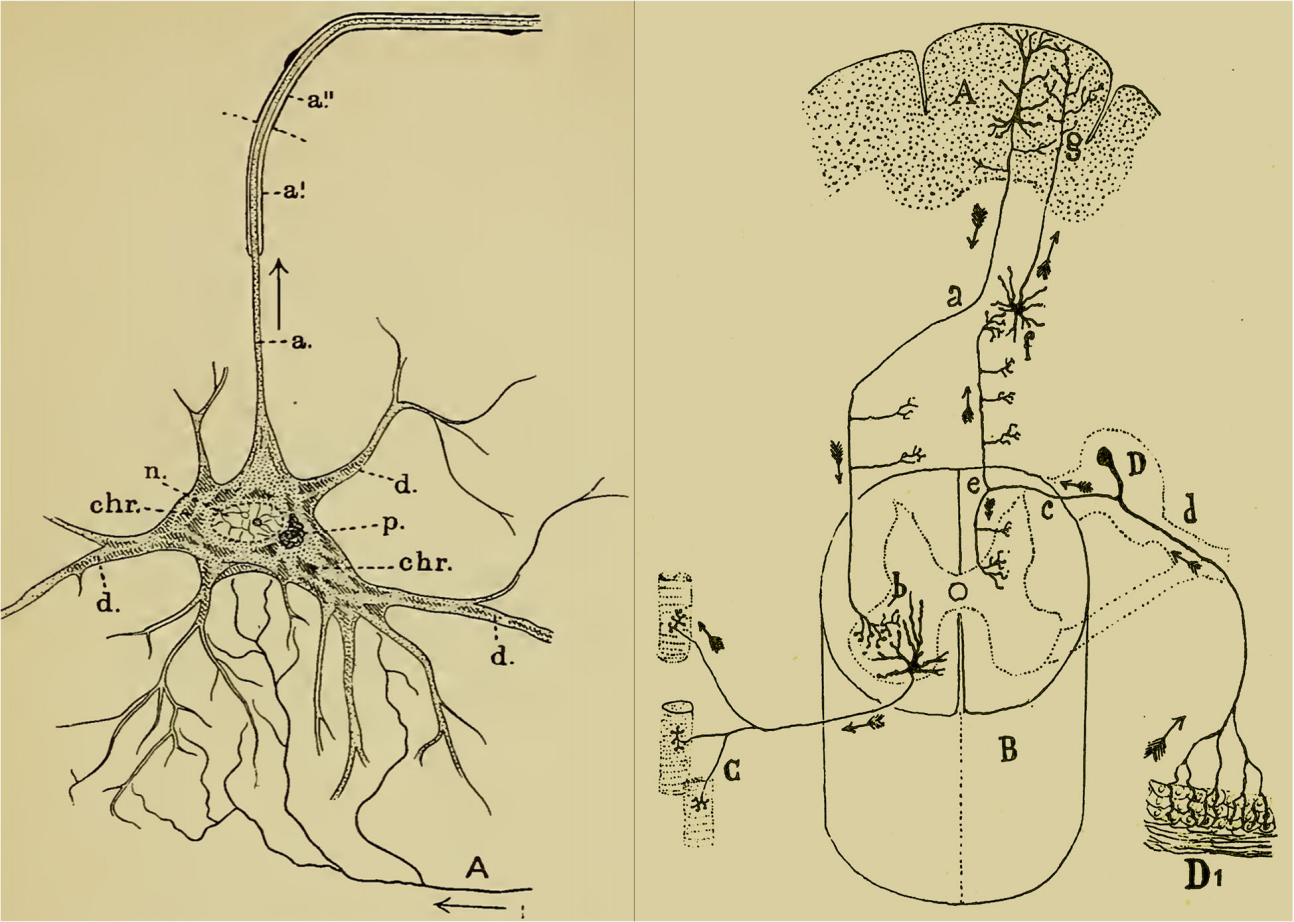
Grand synthesis of structure and function based on Cajal’s law of dynamic polarization. *Left panel*: Sherrington’s definition of synapses was first stated in 1897 and illustrated using this figure in *Foster’s Textbook of Physiology, Part 3*, which he co-wrote [30]. This illustration shows the influence of Cajal’s ideas with an axon drawn (*A*) to terminate in close proximity to the dendrites and soma of a motor neuron and provide information to them (*bottom arrow*). The motor neuron then passes this signal to the periphery via its axon (*upper arrow*). *Right panel*: Four years earlier [31] Cajal provided the connectionist viewpoint for behavior showing how sensory information originating in the skin (*D1*) might pass from one cell to another from spinal cord (*B*) to the cortex (*A*) and back down the spinal cord leading eventually to action as muscle fibers become activated by a motor neuron axon (*C*). This conception is the foundation of the connectomics approach: tracing out all the synaptic connections between neurons

At the end of the 19th century, while Ramon y Cajal was working out his ‘neuron doctrine’ [4], Charles Sherrington was beginning to identify physiological discontinuities in the flow of information that mediated reflexive behaviors which he ascribed to ‘synapsis’ between axons and their targets [5]. Sherrington’s ideas found a strong anatomical correlate in Cajal’s work on the law of dynamic polarization (Fig. 2). It must have been a great aha moment when the worlds of physiology and neuroanatomy seized upon the idea that they were in fact studying the same thing, synapses, from different perspectives. The idea that physical connectivity of neurons could underlie neural function was the grand synthesis of 20th century neurobiology.

Despite the promising beginnings in anatomy and physiology, the conceptual links between cellular connections and behavior have perhaps not evolved as much as one might have hoped. Many modern findings, in fact, seem to emphasize almost the opposite idea: that anatomical connectivity per se is an inadequate platform to understand an organism’s behavior [6]. For example, the fact that synapses can strengthen or weaken or even be silent; the fact that hormonal and paracrine effects can change a neural circuit’s behavior; and the fact that the behavioral state of an organism can change rapidly all suggest that the wiring diagram is insufficient to get at the physical underpinnings of a functioning brain [7].

But these caveats are not the main reason that synaptic networks have not been intensively studied. Rather, for the most part, such data have just not been available. The principal reason is technical: connectional maps of networks require high resolution imaging over large volumes, a challenging mix [8]. However, it appears that neuroscience is on the cusp of entering a time when direct detailed information about network connectivity will be readily available thanks to recent developments in imaging technologies that reveal neural network organization.

**Fig. 3.**
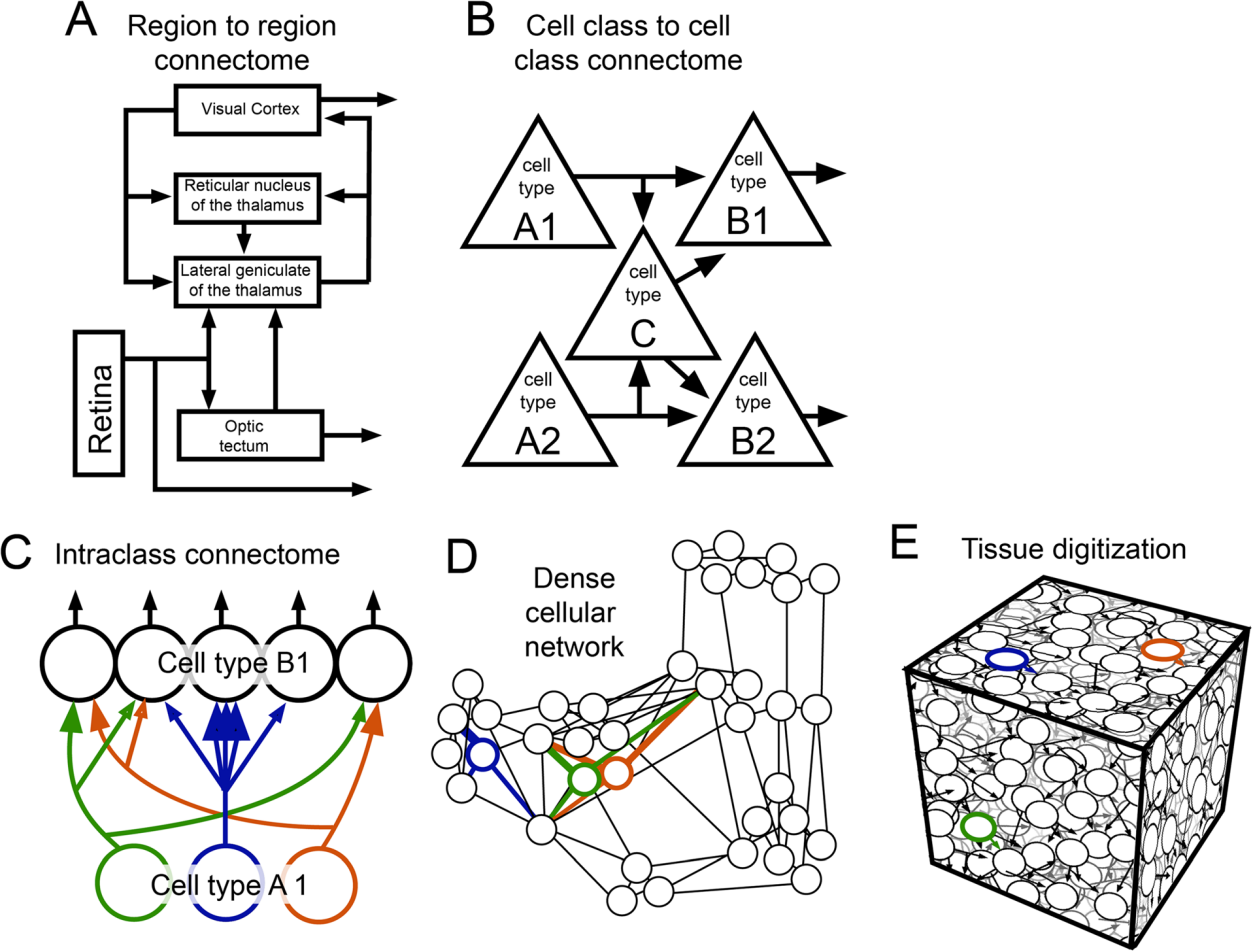
Connectivity maps at different levels of organization. **a** Diagram of region-to-region flow of visual information. **b** Cell type connectivity map illustrates which neuronal types and subtypes innervate which. **c** Intraclass connectome illustrates the patterns of synaptic connections generated by the relative connectivity of individual neurons (colors) of the same type. For example, the *green* and *orange* cell constantly innervate the same targets. **d** Network diagram in which the synaptic connections of multiple populations and subpopulations of neurons can be organized according to their synaptic connectivity. **e** Tissue digitization. An attempt to capture all anatomical connectivity data within a single dataset. Digitization includes ultrastructural description of synapses and relative distribution of synapses across arbors of target neurons

Although connectomics is a nascent field, research is already moving in several different directions. It may be useful to formally divide connectional data mapping into four connectomic categories—projectional, interclass, intraclass, and saturated (Fig. 3)—because these bodies of work are asking quite different kinds of questions, and to some degree require different techniques.

### Projectional connectomics

The brain is unlike other organ systems because the principal cells (neurons) specifically interact with a large number of other cells that may be located considerable distances apart (even meters apart in large animals). Thus, it is essential to map the pathways by which neurons in different parts of the brain are connected. Such long-range connections (Fig. 3a) are most easily mapped by methods that can cover large expanses such as magnetic resonance techniques [9] or labeling by axonal transport [10]. In 2005, the term connectome [11] was coined to refer to a proposed complete mapping of the connectivity matrix of the human brain. Progress on ‘The Human Connectome Project’ has provided a framework for integrating many different kinds of human brain imaging data from many different subjects and has resulted in increasingly more detailed parcellation of human brain regions and their connectivity [12]. Importantly, however, the current limiting resolution of techniques that map full human brains is in the range of a cubic millimeter—a trillion-fold larger than the resolution required of the techniques used to generate maps of synaptic connectivity.

### Interclass connectomics

The brain is also unlike other organ systems because of the sheer diversity of its cellular components. In many animals the matter of neuronal cell diversity is simplified somewhat because it appears that the same neuron class is used multiple times in a single animal’s nervous system. Not only single cell classes, but also multicellular motifs (Fig. 3b) seem to be used repeatedly. The use of stereotyped cellular ensembles is commonplace in all organs (for example, the renal nephron) where the inherent redundancy of multiple copies of the same ensemble improves functional capacity. In the brain, multiple copies seem to play a different role. In the visual system, for example, the same cellular motifs are duplicated many times over in order to analyze each position in visual space. There is nothing redundant about this duplication (damage to a small part of retina leads to a blind spot). However, learning how visual signals are passed from photoreceptors to their downstream targets in one patch of the retina is often sufficient to explain how such signals are processed throughout most of the retina. There is widespread belief—as yet unproven—that a similarly stereotyped circuit might be in use throughout the cerebral cortex.

One challenge in generating and interpreting cell-type connectome data relates to the cell-type classification process per se. Cells belong to multiple overlapping classes depending, in part, on whether the criterion is functional, structural, or biochemical. While we attempt to create logical frameworks by placing things in separate cubby holes, the actual ‘logic’ of animal evolution requires no such tidy classification structure for a nervous system to do its work. The lines between fixed neuronal categories can be especially blurry when the function of a particular neuron is an emergent property that only manifests itself after a protracted period of development and learning [13].

### Intraclass connectomics

Beyond the identification of cell classes and their canonical connectivity is a more subtle problem that is easily seen by considering the connectivity of cerebellar cortex. The cerebellum appears relatively simple: there is only one type of axonal output (from Purkinje cells) and two types of axonal inputs (mossy and climbing fibers). Within the cerebellum there are only a handful of cell types (granule cells, Purkinje cells, and several types of interneurons). The connectivity (in a canonical sense) has been worked out, but both what the cerebellum does exactly and how it does it remain elusive. Why is this? The way the cerebellum works probably depends on how the climbing fibers, parallel fibers, and inhibitory neurons that innervate Purkinje cells are organized. It is not sufficient to know that both classes of axons innervate Purkinje cells. What presumably matters is which particular neurons among each of these classes co-innervate the same Purkinje cell. Understanding this kind of network connectivity is difficult because there may be no intrinsic molecular markers to help discriminate one parallel fiber from any of perhaps hundreds of millions of other parallel fibers in the same cerebellum. Probably some of this connectivity variation is established by the effects of neural activity. Hence, we suspect that it is primarily in the intraclass connectome (Fig. 3c) where one will find the connectional patterns underlying long-term memories.

### Saturated connectomics: a digital brain

A single dataset could contain projectional, canonical, and intraclass connectional information (Fig. 3d) if one were willing (and able) to generate a true digital rendering of a brain containing everything down to every last synapse (or even further to every synaptic vesicle). The important point is that a digital brain (Fig. 3e) with a fully saturated wiring diagram is more useful than an actual brain in a critical way: it can be mined forever by virtue of the conversion of tissue into a permanent digital equivalent.

## From inferring synaptic networks to imaging them

**Fig. 4.**
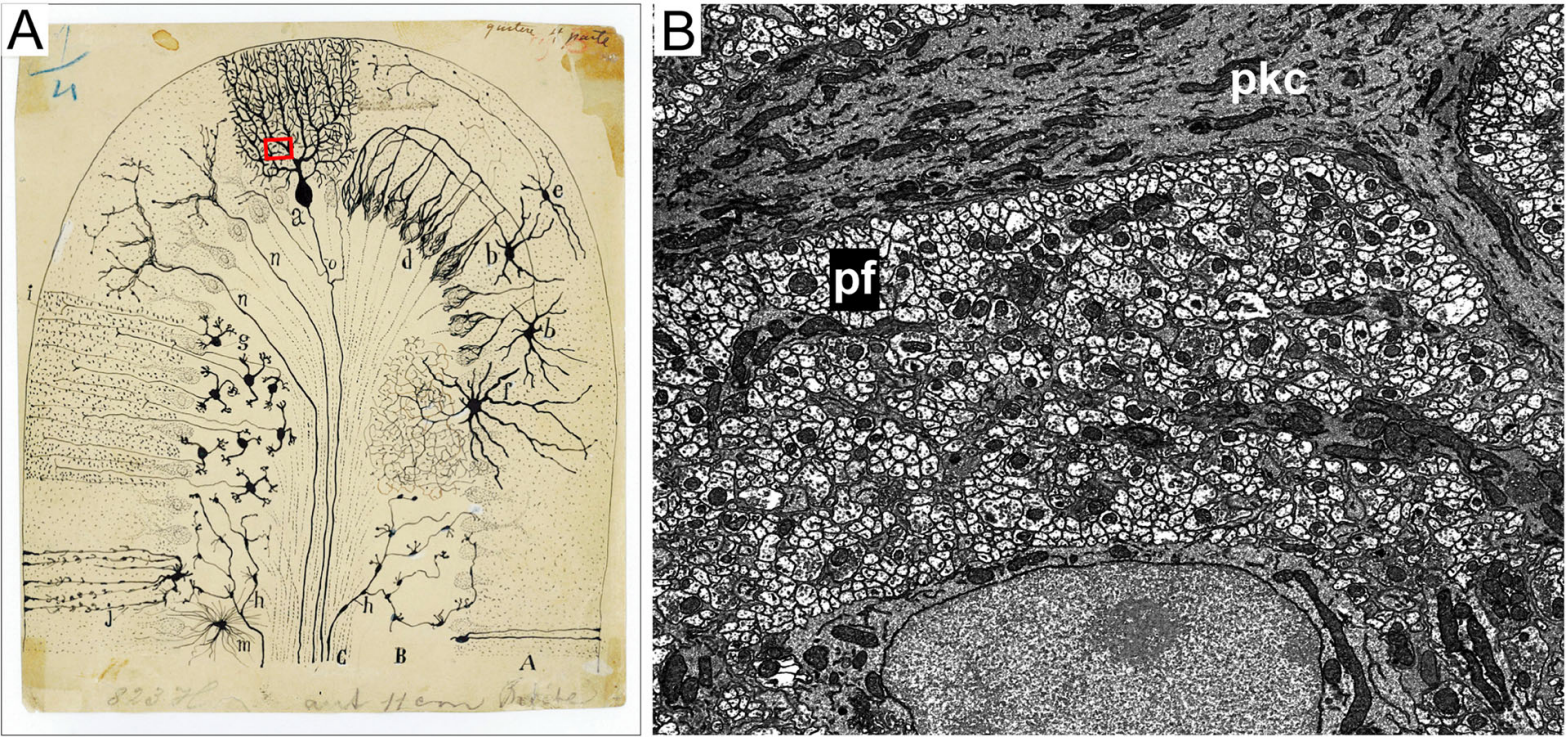
Two views of the cellular organization of the cerebellum. **a** Ramon y Cajal drawing of cell types of the cerebellum based on many observations of Golgi labeled neurons (Cajal Legacy, Instituteo Cajal, Madrid, Spain). *Red box* indicates relative scale of image in panel **b**. **b** Electron micrograph showing density of parallel fibers (*pf*; small, light circular profiles) surrounding the dendrites of a Purkinje cell (*pkc*; dark elongate profiles)

Beginning with Cajal’s use of the Golgi stain, neuroscientists have pursued ever more sophisticated strategies to uncover the neural circuits that underlie functional features of the nervous system. For Cajal the key strategy was using a stain that labeled a small number of the cells in any given piece of tissue. With this tool he was able to identify distinct types of cells by their appearance and, based on close anatomical proximities of axons of one cell type and dendrites and somata of others, he could infer which cell types were likely to be functionally connected. As long as a cell type could be identified from one sample tissue to the next, all the observed connectivities collected from the different tissue samples could be amalgamated into a comprehensive view of how the cell class interacted with cells of other classes. A beautiful Cajal drawing describing the circuitry of a region of the brain is not a single piece of tissue that was observed through his microscope; rather it is a composite of a large number of observations inferentially linked together by commonalities between individual subjects (Fig. 4a).

Despite an expanding array of new labeling and recording approaches, in large part neuroscience still generates maps of synaptic networks based on accretion from observations of a small portion of the network in each of many different samples. The composite circuits are elevated to the status of ‘canonical’ circuits once they seem to have properties that are consistent between individuals and in some cases between species.

## What is missing from canonical circuits?

The trouble with the use of canonical circuits as a descriptor of the way the brain is organized is that the structure of neural tissue is likely not actually canonical. The notion of canonical circuits in important because it provides a list out of the cell types that comprise real circuits and which type is connected to which other type, but we suspect that description is, in most cases, a far cry from an actual neural network. Canonical circuits often ignore the number of different cells of each class that converge on a postsynaptic cell and the number of different target cells of one class that are innervated by an axon. More problematic, they make the implicit assumption that all cells of the same class have the same connectivity so that showing the average connectivity between two cell types is a sufficient surrogate for mapping the ensemble. But what if the differences between the connectivity within cohorts of cells of the same class are important to circuit function? What happens when the synaptic connections of one class of neurons individuate in response to differences in the activity patterns within another class of neurons?

We think the problem with canonical circuit descriptions becomes clear when we consider why nervous systems in many animals (and especially vertebrates) opt for using the same cell type many thousands or even millions of times over. As mentioned above, adding more cells of the same type only increases the computational power of the system if each cell or motif is able to integrate somewhat different pieces of information. This division of computational labor within a class of cells may come about by a largely experience-dependent differentiation in their connectivity. This intraclass circuit diversity simply cannot be captured in canonical views of brain circuits.

While some of the diversity in connectivity between cells of the same class can be produced by random variations or by the topographic distribution of cells relative to potential partners, circuit activity also plays an important role by rewiring neural circuits in development. This network activity-based remodeling is sometimes considered to be a modest refinement occurring at the end of development, as the final opportunity for fine tuning of an otherwise serviceable wiring diagram. But in mammals, at least, the role of activity in affecting wiring may be more central than the notion of fine tuning suggests. Extensive postnatal synaptic remodeling seems fundamental to circuit wiring throughout the nervous system [14–22]. In places like the neuromuscular system, the changes in wiring cannot be considered refinements in that over ~ 90% of the synapses present at birth are eliminated during development [23].

When the connectivity of a neuron is changed as a consequence of the activity of other members of the same neural population, higher order patterns of network connectivity will emerge that cannot be captured by a canonical (type to type) approach to circuit reconstruction. These intraclass patterns of connectivity are only visible when the connectional patterns of many neurons in the same piece of tissue are mapped simultaneously. Critically, maps of many cells in the same piece of tissue are able to reveal the property of contingency: neuron A innervates neuron C only if neuron B does as well. When the intraclass convergence pattern of a dozen or so developing motor neurons is mapped using Brainbow we see a great deal of this kind of contingency wiring (Draft, Turney, and Lichtman unpublished). These ordered connectivity patterns could never be inferred by sampling one or two neurons at time. Thus, to understand how populations of neurons organize into circuits, it is not sufficient to observe one cell’s connectivity and to infer its relationship to a standardized theoretical network. Rather all the cells, or at least many of them, have to be assayed in the same piece of tissue in order to learn the actual network structure of interconnected neurons. To reiterate: it is possible that learned information (such as a particular language) is stored in a way that is invisible at the level of canonical circuits.

## Label everything, image everything

As suggested above, an alternative to the accretion method of reconstructing circuits from many different samples is to *acquire many or all synaptic networks from the same tissue sample*. In the dense neuropil of the central nervous system or the fine processes in invertebrate nervous systems, a complete anatomical description of synaptic connectivity is perhaps most easily obtained with serial electron microscopy (EM; Fig. 4b). The high resolving power of electron microscopes means that tissue can be stained with nonspecific labels that reveal every cell membrane and every organelle. Such nonspecific staining makes electron microscopy fundamentally different from most uses of fluorescence because *all* cells and organelles are visible in the same piece of tissue. Moreover, should one generate a network map of all the cells that are within the volume, such a network map reveals circuit details that cannot be inferred from any number of sparsely annotated data sets [7]. The morphology and ultrastructure of each connected process also provide details that can be used to classify the types and assay the strengths of the connections within the networks.

The ability to see everything comes with a cost: scaling the electron microscopy approach to even moderate sized volumes is non-trivial. With few exceptions, early serial electron microscopy approaches used tissue volumes far smaller than the dendritic arbor of the average mammalian neuron. Many technical obstacles still stand in the way of acquiring large volume EM data sets, from the difficulty of uniformly fixing, staining, and embedding large pieces of tissue to the challenges of imaging large fields of view at suitable resolution in a time scale compatible with the human lifespan (much less the duration of a graduate studentship or postdoc). The problem lies with the joint requirement of large volume and high resolution. In order for small structures, such as synaptic vesicles, fine axon branches, and dendritic spine necks to be visible and traceable, the image voxels (i.e., the three-dimensional equivalent of pixels) must be smaller than the size of these structures. Depending on the technique being used to section and image the tissue, the size of a serial EM voxel ranges from about 500 nm^3^ upwards. In our own experience, circuits and synapses in the mouse brain can be identified and traced with little ambiguity at a resolution of 4 nm × 4 nm × 30 nm [24]. At this resolution, a hundred micron wide cube of tissue (large enough to capture about 50 neuron somata but only parts of a neuronal dendritic arbor) consist of about 2 × 10^12^ voxels (or about 2 terabytes of data), a millimeter wide cube of tissue would consist of 2 × 10^15^ voxels (2 petabytes), a whole mouse brain would be about 2 × 10^18^ voxels (2 exabytes), and a whole human brain would be about 2 × 10^21^ voxels (2 zettabytes). The two terabytes of storage required to hold even the smallest of these volumes is something that was not available for the first 50 years of electron microscopy. Two zettabytes is a substantial portion of the estimated 2.7 ZB digital content of the world and it is probably at least at the moment an unreachable goal.

It is, however, now possible to store and process 100 terabytes or more of digital data (see, for example, [13]). The result is that it seems possible, for the first time, to study synaptic networks of a reasonable sized piece of tissue (hundreds of microns on a side) at any arbitrary resolution from seeing and counting synaptic vesicles at one extreme to seeing whole nervous systems at the other. Of course, acquiring such datasets depends not only on sufficient digital storage, but on many other technical advances that increase the throughput of image acquisition, image processing, and image analysis. New technologies that increase the throughput and reliability of electron microscopy imaging are being developed. For instance, our recent efforts using ATUM-SEM produced approximately one terabyte a day of high resolution EM data for data sets of 100 or so terabytes [13, 25]. This performance is being improved by more than an order of magnitude with newer imaging strategies that could image cubic millimeter (petascale) data sets from start to finish in a matter of months [26].

Exploring these data sets, which consist of trillions of voxels and thousands of image planes, can no longer be accurately described as looking at an image. Rather, one can investigate a piece of digital tissue as if it were an actual brain much the way one can travel anywhere in the digital Earth just with a help of a browser. Importantly the notion that the investigator has seen their entire data set becomes an impossibility. Full resolution images of a cubic millimeter of brain would take more than a century to view.

## Doing biology on digital tissue

In order to turn gray scale digitized tissue into synaptic network data, decisions have to be made about which voxels belong to which cells and which cells form synapses with one another. While human beings are good at this task, a human staring at a 1000 × 1000 square of pixels that changes at a rate of 30 frames per second will be able to view less than a terabyte of data in an 8-hour day. That is, as mentioned already, EM volumes can already be acquired faster than a person could look at all the acquired voxels, much less analyze them. Making use of terabytes of digital tissue therefore requires different image analysis strategies than have been used with biological image data in the past.

## Analyze everything

**Fig. 5.**
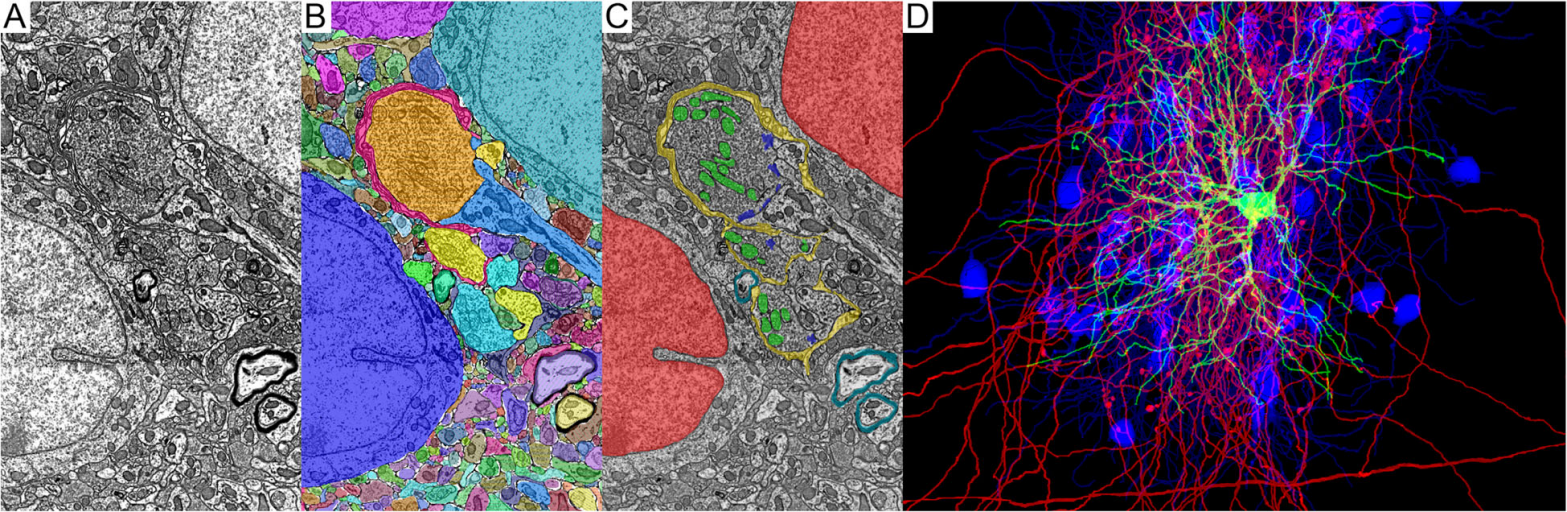
Comparison of saturated vs targeted annotation of three-dimensional EM data sets. The images have not been previously published as such, but are accessible from the datasets [32] published in [13]. **a** Electron micrograph of mouse lateral geniculate nucleus. **b** Saturated annotation of cell identities. Each cell profile from the electron micrograph in panel **a** is assigned a different color. **c** Examples of ultrastructural annotation that form additional layers of information in a saturated annotation of EM volume. *Red* = nucleus, *yellow* = glial encapsulation of synaptic glomerulus, *green* = distinctly light mitochondria of retinal ganglion cells, *blue* = synaptic sites between retinal ganglion cell boutons and thalamocortical neurons, and *cyan* = myelination of axons. **d** Synaptically targeted annotation of three-dimensional EM volume in which seed thalamocortical cell is *green*, the retinal ganglion cells that innervate the seed cell are *red*, and the thalamocortical cells that share retinal ganglion cell innervation with the seed cell are *blue*

Ideally, every voxel of a piece of digital tissue would contain not only the gray value of the raw data, but also annotation data describing to which cell (and subcellular structure) the voxel belongs and, if it is a synapse, some connectivity data (Fig. 5a–c). As mentioned, humans are very good at recognizing cell boundaries, classifying objects, and building connectional maps. Unfortunately, segmenting large data volumes requires recruiting large numbers of people to assign voxels to cellular objects. As newer tools emerge that acquire data at even faster speeds, the cost to hire proportionally more human tracers will be prohibitive. Ultimately, segmentation solutions will need to scale with advances in image acquisition capacity and that will require automated segmentation methods. Methods that in principle will segment every voxel in a digital tissue.

For EM images, current automated segmentation algorithms are computationally demanding and still require human correction of the results. The goal of limiting human editing to almost nothing is still distant, but progress in the field is rapid. As algorithms improve and processing speed gets cheaper, the algorithms should be able to replace the vast majority of human segmentation.

## Targeted exploration

The potential limitations in automatic segmentation bring to light the question of whether it is actually useful to acquire large volumes of digital tissue if only a subset of the image voxels will ultimately be analyzed. Ultrastructural mapping of even a single neuron requires the acquisition of large EM volumes simply because of the long distances traveled by neuronal processes. But if segmenting every voxel within a volume is not practical, targeting reconstruction to a subset of the cells can still yield important biological results. Critically, this approach is distinct from traditional sparse circuit mapping because, after a first round of tracing, additional cells in the same digital tissue can be progressively added to the same network based on their synaptic connectivity.

An example of targeted segmentation would be to first identify a target neuron cell body according to ultrastructural cell typing, antibody labeling, or prior optical characterization of the tissue. The dendritic arbor of the target cell would then be traced and all of its synapses annotated. The boutons on the presynaptic side of each of the synapses would then serve as seed points for the next level of tracing. Tracing these first order connections would reveal the spatial distribution, convergence, and possibly divergence of the relevant axonal inputs of the cell. Each of these traced axons could then be used to identify all of the cells in the tissue that share inputs with the original target cell. By moving through network connectivity in this way, it is possible to learn about the network organization of part of the circuit while only tracing a small percentage of the acquired volume. Once a network of interest is identified within a volume, the morphological and ultrastructural details of its neurons can be characterized. We found this approach, of targeted network tracing and structural characterization, to be effective in mapping the synaptic interactions between visual channels in the mouse thalamus [13] (Fig. 5b, c).

Ultimately, the number of questions that can be asked about a large piece of digital tissue exceeds what can be asked by one scientist or even a single lab. The best use of large EM data sets will, therefore, be achieved by making these datasets easily available so that many researchers can explore them. In particular, maintaining all of the published segmentations in an accessible database will allow questions raised by one round of tracing and publication to be answered in another round. As the automated algorithms improve, more and more of these public databases may well include large-scale automated annotations of the data that can be searched according to morphology and connectivity.

## Reanimating digital tissue

While we have described these new large volume data sets as ‘digital tissue’ it is of course not strictly correct as they are not biologically active. In light of large-scale efforts to create models of functioning neural tissue [27], maybe it would be more accurate to describe these volumes as digital *fixed* tissue. However, if large volumes of neural circuits are annotated, complete with cell morphologies, synaptic connectivity, and ultrastructure, how big of a jump might it be to reanimate this tissue? What will happen when we can apply the functional modeling techniques, currently being used to study statistically inferred neural circuits, to the detailed anatomical structure of an actual neural circuit?

There is considerable debate about how closely such a digitally reconstructed circuit might be able to recreate the behavior of the original circuit [7]. For instance, in order to observe biologically realistic processing in this neural tissue, it will be necessary to provide biologically realistic activity to the circuit’s inputs. However, by constraining the behavior of a large scale anatomically realistic model of a circuit according to the results of large scale imaging of circuit activity and learning rules, we may be able to generate functional circuitry. How closely the behavior of these circuits resembles the functioning of biological circuits will be a critical test of our understanding of neural processing.

## Conclusion

Both cellular and systems neuroscience are making steady progress, but the critical bridge between them, understanding how large numbers of neurons organize themselves into functional networks, is still unbuilt. The transition from being able to image one or two neurons at a time to being able to digitize whole multi-neuronal networks may be the solution. In exploring densely reconstructed networks, we will be able to deal with diversity in the connectivity of neurons not as noise to be averaged out, but as the principal phenomena to be understood.
